# Multiple intravenous tranexamic acid doses in total knee arthroplasty in patients with rheumatoid arthritis: a randomized controlled study

**DOI:** 10.1186/s12891-021-04307-4

**Published:** 2021-05-07

**Authors:** Bing-xin Kang, Hui Xu, Chen-xin Gao, Sheng Zhong, Jing Zhang, Jun Xie, Song-tao Sun, Ying-hui Ma, Xi-rui Xu, Chi Zhao, Wei-tao Zhai, Lian-bo Xiao, Xiao-jun Gao

**Affiliations:** 1 Shanghai University of Traditional Chinese Medicine, Shanghai Guanghua Hospital of Integrated Traditional Chinese and Western Medicine, Shanghai, 200052 CN China; 2 Arthritis Institute of Integrated Traditional Chinese and Western Medicine, Shanghai Academy of Traditional Chinese Medicine, Shanghai University of Traditional Chinese Medicine, Shanghai, 200052 CN China

**Keywords:** Total knee arthroplasty, Rheumatoid arthritis, Tranexamic acid, Blood loss

## Abstract

**Background:**

We aimed to determine the efficacy and safety of multiple doses of intravenous tranexamic acid (IV-TXA) on perioperative blood loss in patients with rheumatoid arthritis (RA) who had undergone primary unilateral total knee arthroplasty (TKA).

**Methods:**

For this single-center, single-blind randomized controlled clinical trial, 10 male and 87 female participants with RA, aged 50–75 years, who underwent unilateral primary TKA were recruited. The patients received one dose of 1 g IV-TXA 10 min before skin incision, followed by articular injection of 1.5 g tranexamic acid after cavity suture during the surgery. The patients were randomly assigned (1:1) into two groups and received an additional single dose of IV-TXA (1 g) for 3 h (group A) or three doses of IV-TXA (1 g) for 3, 6, and 12 h (group B) postoperatively. Primary outcomes were total blood loss (TBL), hidden blood loss (HBL), and maximum hemoglobin (Hb) level decrease. Secondary outcomes were transfusion rate and D-dimer levels. All parameters were measured postoperatively during inpatient hospital stay.

**Results:**

The mean TBL, HBL, and maximum Hb level decrease in group B (506.1 ± 227.0 mL, 471.6 ± 224.0 mL, and 17.5 ± 7.7 g/L, respectively) were significantly lower than those in group A (608.8 ± 244.8 mL, *P* = 0.035; 574.0 ± 242.3 mL, *P* = 0.033; and 23.42 ± 9.2 g/L, *P =* 0.001, respectively). No episode of transfusion occurred. The D-dimer level was lower in group B than in group A on postoperative day 1 (*P* <  0.001), and the incidence of thromboembolic events was similar between the groups (*P* > 0.05).

**Conclusion:**

In patients with RA, three doses of postoperative IV-TXA further facilitated HBL and Hb level decrease without increasing the incidence of adverse events in a short period after TKA.

**Trial registration:**

The trial was registered in the Chinese Clinical Trial Registry (ChiCTR1900025013).

## Background

Rheumatoid arthritis (RA) is an autoimmune disease with erosive arthritis as the main clinical manifestation. The basic pathological manifestations are synovitis, pannus formation, and gradual articular cartilage, and bone destruction, eventually leading to joint deformity and dysfunction [1]. The main onset age for RA is 40–60 years [2].

RA disease activity measures appear to be worse in females than in males [3]. The use of anti-rheumatic drugs and biological agents has delayed the progress of bone destruction in patients with RA; in fact, the number of RA patients receiving total knee arthroplasty (TKA) has gradually decreased over the past few decades. However, in advanced-stage RA patients with severe knee joint destruction, TKA is effective for improving knee function and quality of life [4, 5]. However, TKA can lead to blood loss; the mean blood loss during the perioperative period of TKA can reach 1470 mL [6]. Moreover, RA patients experience an increased incidence of anemia [7]. Low hemoglobin (Hb) levels before surgery also increase the risk of blood transfusion need after surgery [8]. Considering that blood transfusion increases the risk of postoperative infections and prolongs hospital stay [9], it is vitally important to reduce TKA perioperative blood loss and accelerate postoperative recovery. In previous studies, the perioperative use of multi-mode blood management successfully reduced perioperative blood loss [10, 11]; a combination of multiple strategies of blood management also reduced the total blood transfusion rate after TKA to less than 4% [12].

Postoperative bleeding is mainly caused by fibrinolysis due to surgical trauma. Although tourniquet application can reduce intraoperative bleeding, fibrinolysis and postoperative bleeding will increase following the postoperative release of the tourniquet [13]. The anti-fibrinolytic drug tranexamic acid, by preventing the combination between plasminogen and fibrin, protects fibrin from degradation by plasmin to achieve hemostasis [14]. Clinical studies have confirmed that tranexamic acid can reduce the incidence of anemia after TKA and reduce transfusion rate without elevating the incidence of venous thrombosis [15–18]. A meta-analysis showed that 2 g tranexamic acid had the best effectiveness and safety profile [19]. However, there is still no consensus on the optimal dose and timing of tranexamic acid administration [20].

Patients with RA may experience mild to moderate anemia; thus, they have a higher risk of infection at the surgical site than that in patients with OA [21]. We hypothesized that high drug doses will be beneficial for RA patients undergoing TKA. In our orthopedic department, enhanced recovery after surgery is strongly advocated, for which blood management is essential. In this randomized controlled trial, the pharmacokinetics of tranexamic acid were determined to evaluate the efficacy and safety of multi-dose intravenous tranexamic acid (IV-TXA) in alleviating postoperative blood loss in patients with RA who underwent primary unilateral TKA.

## Methods

### Study design

This was a single-center, single-blind, randomized controlled trial. The study was conducted at the Department of Orthopedics in Shanghai Guanghua Hospital of Integrated Traditional Chinese and Western Medicine and registered in the Chinese Clinical Trial Registry (ChiCTR1900025013). The study was approved by the institutional review board (Ethics Committee of Shanghai Guanghua Hospital of Integrated Traditional Chinese and Western Medicine). All experimental procedures were conducted according to the Standards of Reporting Trials (CONSORT) recommendations for randomized controlled trials [22], and all participants provided written informed consent before enrolment.

### Sample size calculation

The sample size was calculated based on the amount of hidden blood loss (HBL) during tranexamic acid therapy. The overall standard deviation was σ = 250, and the allowed error estimate was δ = 200. These values were estimated using the statistical following formula ${n}_1={n}_2=2\times {\left[\frac{\left({Z}_{a/2}+{Z}_{\beta}\right){/}_{\sigma }}{\delta}\right]}^2$. Predicting an estimated dropout rate of 10%, 104 subjects would be required to yield a statistical power of 90% with a significance level of 0.05.

### Patients

From September 2019 to May 2020, we screened patients aged 50–75 years who underwent primary unilateral TKA for RA, using doppler ultrasound and computed tomography examination to identify patients without deep vein thrombosis (DVT) and pulmonary embolism (PE), respectively. The exclusion criteria included a diagnosis of other types of arthritis (i.e., not RA), renal dysfunction, or severe cardiovascular or cerebrovascular diseases; patients who reported prolonged use of oral anticoagulant drugs were also excluded. The elimination criteria included acquired color vision disorder, active intravascular coagulation patients, and a history of seizures.

### Randomization and drug administration

All eligible patients were randomized into two groups using computer-generated randomization by a statistician who was not involved in the trial. The group data were saved by the statistician. The allocation was concealed in consecutively numbered, sealed, opaque envelopes. IV-TXA (1 g) was administered 10 min before skin incision by an anesthesiologist, and tranexamic acid (1.5 g dissolved in 10 mL of normal saline) was administered via articular injection by a surgeon after cavity suture during the surgery. Patients in group A received one dose of IV-TXA (1 g) 3 h postoperatively, while those in group B received three doses of IV-TXA (1 g) 3, 6, and 12 h postoperatively. The doses were administered by a nurse after surgery. The surgeon, anesthesiologist, and statistician were blinded to the trial. Only the nurses knew the patients’ group assignment. Tranexamic acid was produced from Hunan Dongting Pharmaceutical and used according to the second edition of the 2015 Chinese Pharmacopoeia and Drug Supplement Application Approval (2013B02016), YBH07372010, under the National Drug Standard approval number H43020565.

### Perioperative anti-rheumatic treatment

Methotrexate and hydroxychloroquine were used during the perioperative period. Leflunomide was discontinued at 1 week before surgery. The use of other disease-modifying anti-rheumatic drugs was discontinued 2 days before surgery and restarted at 1–2 days after gastrointestinal function recovery. The use of newer biologic agents, such as tumor necrosis factor-alpha, was discontinued at four to five half-lives before surgery and restarted after wound healing and elimination [23, 24].

### Preoperative Anemia treatment

Patients with preoperative Hb level < 12 g/dL were administered erythropoietin 10,000 U orally once a day; the Hb level was checked regularly, and the administration was stopped when Hb level reached > 15 g/dL.

### Surgical procedure and postoperative management

For perioperative prophylaxis, cefazolin sodium antibiotics were administered 30 min before and 24–48 h after surgery. General anesthesia was administered by an anesthetist. Blood pressure was controlled to within 80–100 mmHg/60–70 mmHg throughout the procedure. Tourniquet was inflated to 100 mmHg above the systolic pressure before incision and deflated after incision closure. The surgery was performed by a single surgeon using the same technique. All patients received a surgeon-selected, cemented, posterior-stabilized prosthetic design with patellar resurfacing. Drains and blood salvage after surgery were not performed for these patients. Postoperatively, the elastic bandage was compressed, and the operated limb was bandaged for 24 h. The patients were discharged on post-operative day (POD) 14 provided they met the discharge criteria. The discharge criteria included well-healed wound, no wound leakage, no infection, no swelling, no pain, and normal body temperature, with the knee joint able to be activity flexed by more than 90 degrees; Hb level > 70 g /L was an additional criterion.

During hospitalization, all patients received physical prophylaxis and chemoprophylaxis for venous thromboembolism. The patients were asked to perform equal-length contractions of the femoral quadriceps, ankle pump movements, lower-extremity strength training, and motion exercises on the day after surgery. At 6 h after surgery, perioperative oral rivaroxaban (10 mg, once a day for 14 days) was prescribed to prevent thrombosis [25]. Blood transfusions was administered to patients with postoperative Hb level of less than 70 g/L or any organ dysfunction related to anemia regardless of Hb level [26].

### Outcome measures

Perioperative hematocrit (Hct) and Hb levels, coagulation index, and renal function were measured preoperatively and on POD 1, 3, 7, and 14.

The Nadler formula [27] was used to estimate patient blood volume (PBV), and the Gross [28] formula was used to calculate blood loss based on PBV and Hct level reduction.

Intraoperative blood loss (IBL) was estimated based on the amount of liquid in the negative pressure drainage bottle + the amount of liquid in the gauze – the amount of saline. a piece of soaked gauze contained approximately 20 mL of the liquid.

PBV = K1 × height^3^ (m^3^) + K2 × weight (kg) + K3. For males: K1 = 0.3669, K2 = 0.03219, K3 = 0.6041. For females: K1 = 0.3561, K2 = 0.03308, K3 = 0.1833. Total red blood cell loss (TBL) = PBV × (Hct_pre_ – Hct_post_)/Hct_ave_, where Hct_pre_ = initial pre-operative Hct level, Hct_post_ = lowest Hct postoperative, Hct_ave_ = average of Hct_pre_ and Hct_post_. HBL is defined as TBL minus IBL plus transfusion. Thus, HBL = TBL – IBL + transfusion.

The follow-up time was 14 days after surgery. Patients were monitored for adverse events (e.g., DVT, PE, wound complications, infection, and acute renal failure). Transfusion rate and adverse events were assessed postoperatively during the inpatient hospital stay.

### Statistical analysis

Analyses were performed using SPSS Version 25.0 (IBM Corp., Armonk, New York). Data of continuous variables were evaluated for normal distribution using the Shapiro-Wilk test and presented as means ± standard deviations (SDs). Differences between groups were compared using two independent sample *t*-tests. Pearson’s chi-square test was used to analyze categorical variables. *P*-values of less than 0.05 indicated statistical significance.

## Results

### Patient characteristics

**Fig. 1:**
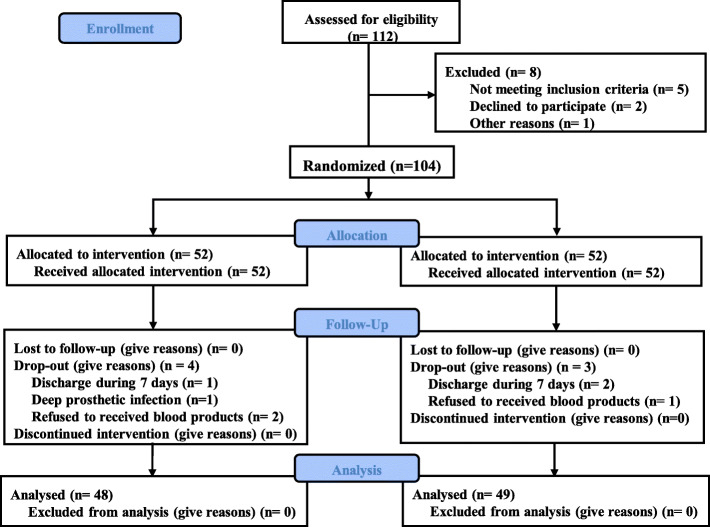
Consolidated Standards of Reporting (CONSORT) flow diagram. Name of the registry: Clinical observation of multiple dose administration of tranexamic acid in patients with rheumatoid arthritis after total knee arthroplasty. Prospective registration, ChiCTR1900025013. Registered 7 August 2019,

Between September 2019 and May 2020, a total of 104 participants were assessed for eligibility. They were randomized homogeneously into two groups (52 in group A and 52 in group B) to receive the study medication. The duration of follow-up was 2 weeks. A total of 7 patients were drop-out owing to the following reasons: 3 patients were discharged within 10 days; 1 had an infection, and 3 refused to receive blood products (Fig. 1). Patient demographics and per- and intra-operative variables were compared between the two groups (Table 1**)**.

**Table 1:** Preoperative and intraoperative characteristics of the patients

Variable Mean ± SD	Group A (***n*** = 48)	Group B (***n*** = 49)	***P***-value
Patient characteristics			
Age (y)	66.4 ± 5.9	66.5 ± 5.5	0.921^a^
Gender (male/female),	4/44	6/43	0.529^b^
Body mass index (kg m^−2^)	21.8 ± 3.4	22.4 ± 3.1	0.397^a^
Patient blood volume (mL)	3487.7 ± 512.9	3525.3 ± 520.5	0.721^a^
Preoperative laboratory values			
Hematocrit (%)	36.1 ± 3.2	36.9 ± 3.7	0.215^a^
Hemoglobin (g/L)	117.5 ± 13.9	119.2 ± 14.8	0.565^a^
Platelets (× 10^9^/L)	232.6 ± 60.6	226.3 ± 60.2	0.605^a^
D-dimer (mg/L)	1.1 ± 0.6	1.0 ± 0.5	0.623^a^
Activated partial thromboplastin time (s)	26.3 ± 4.0	25.9 ± 3.6	0.685^a^
Fibrinogen (g/L)	3.6 ± 0.9	3.7 ± 1.1	0.608^a^
Prothrombin time (s)	11.5 ± 0.8	11.3 ± 0.6	0.191^a^
International normalized ratio	1.0 ± 0.1	1.0 ± 0.1	0.294^a^
Erythrocyte sedimentation rate (mm/h)	38.0 ± 18.8	37.1 ± 19.8	0.819^a^
C-reactive protein (mg/L)	11.2 ± 7.3	11.4 ± 6.4	0.902^a^
Intraoperative blood loss (mL)	34.8 ± 6.8	34.5 ± 7.1	0.842^a^

^a^Two independent sample t tests

^b^Pearson’s chi-square test

### Blood loss, maximum Hb drop, and transfusion rate

The decrease in the mean TBL, HBL, and Hct and Hb levels was lower in group B than in group A. The D-dimer levels was lower in group B than in group A on POD 1. For-three-patients in group A and 40 patients in group B received EPO. There was on significant difference between the group. None of patients received a blood transfusion during the follow-up period (Table 2).

**Table 2:** Primary and secondary outcomes of regarding laboratory values after surgery

Variable Mean ± SD	Group A (***n*** = 48)	Group B (***n*** = 49)	***P***-value^*^
Primary outcomes			
Total red blood loss (mL)	608.5 ± 239.9	506.1 ± 227.0	0.038
Hidden red blood loss (mL)	571.0 ± 237.3	471.6 ± 224.0	0.036
Maximum hemoglobin drop	23.7 ± 9.4	17.5 ± 7.7	< 0.001
Secondary outcomes			
Transfusion (%)	0	0	–
Postop. laboratory values			
Hematocrit (%)			
POD 1	32.8 ± 2.8	34.1 ± 3.5	0.040
POD 3	30.4 ± 2.6	32.5 ± 3.4	0.001
POD 7	31.8 ± 3.1	33.5 ± 3.5	0.014
POD 14	33.8 ± 2.8	34.4 ± 3.0	0.346
Hemoglobin (g/L)			
POD 1	105.0 ± 9.3	108.7 ± 12.4	0.108
POD 3	94.2 ± 9.3	102.2 ± 11.8	< 0.001
POD 7	101.5 ± 10.6	106.8 ± 12.3	0.024
POD 14	109.2 ± 8.8	110.4 ± 10.2	0.542
D-Dimer (mg/L)			
POD 1	5.5 ± 2.9	1.0 ± 0.5	< 0.001
POD 3	3.8 ± 1.8	3.8 ± 2.1	0.998
POD 7	3.6 ± 1.4	3.1 ± 1.4	0.085
POD 14	2.9 ± 1.2	2.7 ± 1.2	0.326

^*^ Two independent sample t tests. *POD1* post-operative day 1, *POD3* post-operative day 3, *POD7* post-operative day 7, *POD14* post-operative day 14

### Complications and adverse events

All incisions were healed by the first intention, and no patient developed DVT, PE, acute renal failure, or other adverse events. There were no statistically significant differences in calf vein thrombosis and superficial infection between the two groups (*P* > 0.05; Table 3).

**Table 3:** Complications

Variable	Group A (***n =*** 48)	Group B (***n =*** 49)	***P***-value
Deep vein thrombosis	0	0	
Pulmonary embolism	0	0	
Calf muscular vein thrombosis	3	4	0.717^a^
Superficial infection	1	0	0.312^a^
Deep prosthetic infection	0	0	
Shock	0	0	
Cardiac infarction	0	0	
Wound complications	0	0	
Acute renal failure	0	0	

^a^Pearson’s chi-square test

## Discussion

Our results revealed the effectiveness of three regimens of tranexamic acid therapy in reducing postoperative HBL in RA patients. The stress-induced damage of peripheral blood vessels caused by operation and the use of a tourniquet during operation promotes the occurrence of postoperative fibrinolysis and increases the amount of HBL due to the blood lost into the tissue intraoperatively and postoperatively, accounting for approximately 50% of the TBL [29]. With the reduction of the tourniquet, the fibrinolysis around the wound reached a peak within 6 h and was maintained for 18 h [30]. The half-life of tranexamic acid in plasma is 2–3 h [14], and its antifibrinolytic effect is maintained for approximately 8 h [31]; after intravenous administration, its 24 h recovery from urine is approximately 90% [14]. We repeated IV-TXA three times postoperatively to ensure that the concentration of TXA in the plasma was maintained. Based on in vivo and in vitro data, the effective therapeutic plasma concentration of tranexamic acid in inhibiting fibrinolysis has been determined to be 5–10 mg/L or 10–15 mg/L [31, 32].

Considering the pharmacological characteristics of tranexamic acid, a single dose of IV-TXA (1 g) after surgery may not achieve the maximum antifibrinolytic effect. Although it has been reported that tranexamic acid does not reduce the rate of blood transfusion after joint replacement in patients with RA [33], we investigated three postoperative doses of IV-TXA (1 g). Our results have shown that the concentration of blood and antifibrinolytic effect of tranexamic acid were maintained during the whole process of fibrinolysis. Decrease in HBL as well as Hct and Hb levels during hospitalization were lower with multiple doses than with a single dose. Hct and Hb levels in both groups decreased to the lowest level on POD 3, indicating that HBL persisted 3 days after surgery.

Multiple strategies have been developed to reduce blood loss in the perioperative period, including preoperative anemia assessment, minimally invasive surgery, shortening of the operation time, use of antifibrinolytic drugs, and postoperative nutritional supplement. Consequently, the number of patients requiring blood transfusion after the operation has been decreasing.

Many studies have shown that repeated administration of postoperative tranexamic acid (mainly in OA patients) is not associated with an increased risk of venous thromboembolic events [16, 34, 35]. Tzatzairis et al. [16] showed that during TKA without the use of tourniquet, three doses of perioperative IV-TXA (15 mg/kg) reduced blood loss, Hb level decrease, and transfusion rate, leading to faster rehabilitation. Sun et al. [36] showed that the administration of a total dose of 30 mg/kg tranexamic acid preoperatively combined with the administration of tranexamic acid twice postoperatively was more effective in reducing postoperative blood loss. Lei et al. [18] conducted a randomized controlled trial involving 159 patients and claimed that a five-dose regimen could further reduce the blood loss, minimize inflammation, enhance mobility, and shorten the length of hospital stay, without increasing the risk of DVT and PE. Maniar and colleagues [7] conducted a prospective randomized controlled trial in 240 patients and claimed that a three-dose regimen was more successful than a single-dose regimen, and a two-dose administration could be the least effective regimen in reducing TBL.

To the best of our knowledge, this is the first randomized controlled study to investigate the efficacy of multi-dose tranexamic acid after TKA in reducing postoperative HBL in patients with RA. Although our results differ from those of Good et al. [37], we believe that a postoperative adequate dose of tranexamic acid administered at least three times can effectively reduce HBL after surgery.

Unlike OA patients, RA patients highly express high levels of inflammatory factors, such as IL-1, IL-6, and TNF- α, which leads to the upregulation of procoagulant factors and downregulation of anticoagulation factors [38]. Thus, repeated postoperative administration of IV-TXA may increase the incidence of DVT and PE. We evaluated the occurrence of DVT and PE by observing the clinical symptoms of patients, D-dimer levels, Doppler ultrasound data, and pulmonary computed tomography data. The results showed that the average level of D-dimer in group A was significantly higher than that in group B within 1 day after surgery, indicating that three doses of postoperative IV-TXA could fibrinolysis and inhibit fibrinolysis effectively, thereby reducing HBL after surgery.

The D-dimer level reflects changes in blood coagulation and fibrinolysis in the body. An increase in D-dimer level is an indicator of hypercoagulability and hyperfibrinolysis and a preferred index for determining the occurrence of DVT [39]. D-dimer levels can increase under trauma, inflammation, and surgery [40]. Conventionally, D-dimer level of higher than 0.5 mg/L is the cut-off value for the diagnosis of venous thrombosis; however, D-dimer level naturally increases with age, and in patients aged more than 50 years, the D-dimer cut-off value is defined as the patient’s age times 10 μg/L [41]. The occurrence of venous thrombosis can be safely ruled out in patients with no symptoms of venous thrombosis, as their D-dimer level is lower than normal [42]. The determination of D-dimer level is recommended for patients with a low or moderate clinical probability of DVT [43].

According to the Chinese expert guidelines for the prevention of venous thrombosis after TKA, anticoagulants should be used for at least 10–14 days, and postoperative lower limb functional rehabilitation training should be performed to prevent the occurrence of venous thrombosis [44]. Studies have shown that under the contemporary prophylactic regimens, the incidence of DVT and PE after TKA is very low [45], the top-ranking intervention for preventing DVT being rivaroxaban [46].

A tourniquet can decrease intraoperative bleeding and facilitate bone-prosthesis adhesion, but it can also increase HBL after operation [47]. Some studies have also shown that a tourniquet increases the degree of pain for a short time after surgery, but it does not increase the recovery time of knee function after TKA [48]. We used a tourniquet during operation and administered multi-dose tranexamic acid antifibrinolytic therapy after the operation. Our results showed that our perioperative blood management program can achieves a balance between bleeding and hemostasis as well as anticoagulation and anti-fibrinolysis effects.

There are, however, some limitations to the present study. Firstly, due to the incidence of RA in females being higher than that in males, there was an uneven male to female ratio; there were more female than male patients in the study. We plan to conduct future clinical studies to reduce the impact of sex on the study results. Second, owing to postoperative blood loss and ethical considerations, we did not establish a placebo group to evaluate the effectiveness of tranexamic acid. Third, the examination period of postoperative outcomes was not strictly 24 and 72 h after operation; thus, we are planning to design another study to obtain more accurate results. Fourth, functional recovery of the knee after surgery was not evaluated in this study. Finally, we performed only lung CT to examine patients with symptoms to determined whether there was PE. Although the half-life of tranexamic acid is short, the short follow-up time may not be adequate to fully assess the risk of DVT and other complications after multiple doses of IV-TXA in patients with RA; extending the follow-up period will be considered in future studies.

## Conclusion

This prospective, randomized controlled trial based on multi-strategic blood management revealed that three doses of IV-TXA administered postoperatively reduced HBL and Hb level decrease, but it caused no adverse events within a short period of time after TKA in patients with RA.

## Data Availability

The datasets used and analyzed during the current study are available from the corresponding author on reasonable request.

## Abbreviations

IV-TXA: Intravenous tranexamic acid
RA: Rheumatoid arthritis
TKA: Total knee arthroplasty
TBL: Total blood loss
HBL: Hidden blood loss
Hb: Hemoglobin
Hct: Hematocrit
DVT: Deep vein thrombosis
PE: Pulmonary embolism
POD: Post-operative day
